# Predictive Value of Preoperative Positive Urine Cytology for Development of Bladder Cancer After Nephroureterectomy in Patients With Upper Urinary Tract Urothelial Carcinoma: A Prognostic Nomogram Based on a Retrospective Multicenter Cohort Study and Systematic Meta-Analysis

**DOI:** 10.3389/fonc.2021.731318

**Published:** 2021-10-01

**Authors:** Bo Fan, Yuanbin Huang, Shuang Wen, Qiliang Teng, Xinrui Yang, Man Sun, Tingyu Chen, Yan Huang, Yumei Wang, Zhiyu Liu

**Affiliations:** ^1^ Department of Urology, Second Affiliated Hospital of Dalian Medical University, Dalian, China; ^2^ Department of Clinical Medicine, Dalian Medical University, Dalian, China; ^3^ Department of Pathology, Dalian Friendship Hospital, Dalian, China; ^4^ Department of Urology, Cancer Hospital of China Medical University, Liaoning Cancer Hospital and Institute, Shenyang, China; ^5^ Department of Clinical Laboratory, Second Affiliated Hospital of Dalian Medical University, Dalian, China

**Keywords:** upper urinary tract urothelial carcinoma, preoperative urine cytology, intravesical recurrence, cohort study, nomogram, meta-analysis

## Abstract

**Background:**

Upper urinary tract urothelial carcinoma (UUT-UC) is a rare and severe urinary malignancy. Several studies have explored the relationship between preoperative urine cytology and intravesical recurrence (IVR) in patients with UUT-UC. However, the results of these studies are controversial or even contradictory, and investigations with UUT-UC patients in northeast China are rare.

**Methods:**

We first estimated the prognostic significance of preoperative urine cytology in the outcomes of intravesical recurrence in 231 UUT-UC patients (training cohort = 142, validation cohort = 89) after radical nephroureterectomy (RNU) by the nomogram model. Subsequently, we quantitatively combined our results with the published data after searching several databases to assess whether preoperative positive urine cytology was associated with poor intravesical recurrence-free survival and a high risk of tumor malignant biological behavior.

**Results:**

Firstly, the multicenter retrospective cohort study demonstrated that preoperative positive urine cytology correlated with poor intravesical recurrence-free survival and can serve as significant independent predictors of IVR by Kaplan–Meier curves and Cox regression analysis. The construction of the nomogram demonstrated that predictive efficacy and accuracy were significantly improved when preoperative urine cytology was combined. Meanwhile, meta-analysis showed that preoperative positive urine cytology was associated with a 49% increased risk of IVR. In the subgroup analysis by region, study type, and sample size, the pooled hazard ratios (HRs) were statistically significant for the Japan subgroup (HR 1.32), China subgroup (HR 1.88), cohort study subgroup (HR 1.45), and the single-arm study subgroup (HR 1.63).

**Conclusions:**

Preoperative urine cytology was validated as a potential predictor of intravesical recurrence in patients with UUT-UC after RNU, although these results need to be generalized with caution. Large, prospective trials are required to further confirm its significance in prognosis and tumor malignant biological behavior.

## Introduction

Upper urinary tract urothelial carcinoma (UUT-UC), which runs from the renal pelvis and calyces to the distal ureter, is widely acknowledged to be a urinary malignancy that originates from the urothelium. UUT-UC accounts for 5%–10% of all urothelial carcinomas, and it is usually considered as a rare urothelial tumor. The average prevalence is 1–2 per 100,000 in the United States (1, 2). UUT-UC is more commonly diagnosed in Asian countries, such as China (including mainland and Taiwan), and can even reach 20–30 cases per 100 persons in high-incidence areas (3). Although the industry-recognized standard treatment for UUT-UC is radical nephroureterectomy (RNU) with bladder cuff excision, several authors have investigated whether subsequent bladder cancer occurrence is highly common after the management of UUT-UC. During follow-up, approximately 22%–50% of UUT-UC patients who undergo RNU have bladder cancer recurrence; additionally, the recurrence rates at 5 years can reach more than 30% (4–7). Recurrence and rapid progression of bladder cancer after UUT-UC significantly increase the psychological anxiety and financial burden of patients and even lower their quality of life (8).

Previously, controversial predictive factors may result in poor prognosis for UUT-UC and intravesical recurrence, such as patient-specific factors (age, sex, diabetes mellitus, or history of bladder cancer), tumor-related factors (location, size, T stage, or architecture), and environmental factors (smoking, long-term use of aristolochic acid, etc.) (8–12). It is worth noting that the 2-year recurrence-free survival rate is less than 56% for stage ≥pT1 cancer, and the 5-year specific survival rate is less than 50% for stage ≥pT2 cancer (13). Therefore, exploring the correlation between various predictors and intravesical recurrence in patients with UUT-UC after RNU plays a crucial role in comprehensive clinical prevention and treatment. Such work depends on many types of diagnostic techniques (14).

As a bladder cancer diagnosis and follow-up method, urine abscission cytology has been used for many years. In 1864, Samders discovered cancer cells in the urine of bladder cancer patients. Since then, urine cytology has become a diagnostic tool for bladder cancer (15, 16). Urine samples are used by researchers to obtain high-quality diagnostic results because of their characteristics. With the advancement of immunology and molecular biology methods, many bladder tumor markers have been found over time. These markers have clinical application value for the early diagnosis and treatment of bladder cancer (17). Cystoscopy and urine cytology have already become standards for the diagnosis and follow-up of bladder cancer. Previous studies have shown that urine cytology correlates with the prognosis of bladder cancer (18–20).

Although studies have focused on urine cytology in the prognosis of intravesical recurrence in UUT-UC patients who underwent RNU (21, 22), the number of studies in China is limited (23, 24). Moreover, evidence is lacking on the current role of this modality. To draw a persuasive conclusion, we first conducted a training cohort (*n* = 142) and a validation cohort (*n* = 89). We collected clinical data from UUT-UC patients who underwent RNU to retrospectively evaluate the prognostic value of urine cytology and intravesical recurrence in patients with UUT-UC in China. We then combined related prognostic factors of UUT-UC with preoperative urine cytology and provided a nomogram model for individualized clinical diagnosis, treatment, and follow-up. Additionally, we conducted a meta-analysis to systematically explore the relationship between preoperative urine cytology and intravesical recurrence in patients with UUT-UC. Similarly, we further aimed to adequately determine the impacts of preoperative urine cytology for predicting oncological progression in UUT-UC, including high-grade tumors, muscle invasion, and lymphovascular invasion.

## Materials and Methods

### Histopathological Evaluation

Standardized pathological protocols were adopted to evaluate all the specimens for urine cytology. We collected as much fresh midstream urine as possible in the morning, extracted the suspension, and smeared it on the slides. Two or three slides were generated for each sample. Hematoxylin and eosin staining was performed for 5 and 3 min, respectively, and then a synthetic resin was used to mount the slides. All specimens were reviewed by urological pathologists. To avoid bias, the samples were blinded.

### Study Population

Prior to this study, the information collection and experimental procedures of this study were approved by the institutional review board. In total, from January 2008 to December 2018, medical records of UUT-UC patients who underwent RNU at three medical centers in northeast China, namely, the Second Affiliated Hospital of Dalian Medical University, Affiliated Dalian Friendship Hospital of Dalian Medical University, and Cancer Hospital of China Medical University, were examined for our retrospective study. Ethical approval was granted by the Ethical Review Board of the Second Affiliated Hospital of Dalian Medical University, Cancer Hospital of China Medical University, and the Affiliated Dalian Friendship Hospital of Dalian Medical University. It is worth emphasizing that patients with the following several conditions were excluded from the normative samples: 1) history of prior urothelial carcinoma in the bladder, 2) urothelial carcinoma in both the upper urinary tract and bladder, 3) undergoing attendant cystoprostatectomy, and 4) history of neoadjuvant chemotherapy or radiotherapy. Finally, a total of 231 eligible patients were sequentially included. The training cohort included 142 patients with 83 patients from the Cancer Hospital of China Medical University and 59 patients from the Affiliated Dalian Friendship Hospital of Dalian Medical University. Meanwhile, the validation cohort included 89 patients from the Second Affiliated Hospital of Dalian Medical University (25–27). We extracted the following parameters from the hospital medical records: sex, age, chief complaint, tumor traits (site, stage, grade, laterality, location, and focality), lymph node status, surgery type, and adjuvant chemotherapy. Tumor stage was assessed by the 2002 TNM classification of the American Joint Committee on Cancer, and the 1998 WHO/International Society of Urologic Pathology consensus classification was used to evaluate the grades of tumors.

### Preoperative Evaluation of Urine Cytology

According to the judgment of the physician, urine samples for cytological examination were collected one to three times. The specimens with preoperative positive urine cytology could be detected by the following findings: 1) malignancy voided urine cells, 2) suspicious voided urine cells, and 3) both (28, 29). Other cases were all deemed negative samples. Positive cytology indicated malignancy, while negative cytology indicated benign or normal tissue (30). The final judgment was confirmed by two pathologists who were blinded to the clinical outcomes.

### Follow-Up and Surveillance Regimen

In the first year after the RNU operation, observations were made every 3 months. From the second year to the fifth year, the frequency was slowed down to every 6 months and once per year thereafter. The measurements of surveillance include serum chemistry studies, routine urine and blood examinations, cystoscopy, and various imaging examinations, such as radiography or CT of the urinary tract. In addition, the physicians responsible for non-surviving patients identified the causes of death and then issued chart reviews or death certificates.

### Statistical Analysis

The association between preoperative urine cytology and clinicopathological parameters was appraised using Fisher’s exact test and the chi-squared test. To directly reflect the degree of bladder recurrence after RNU, intravesical recurrence-free survival (IV-RFS), which was defined as the death rate caused by recurrence of UUT-UC after RNU from a recurrent lesion located in the bladder, was used to statistically compare the different groups by the log-rank test. Taking various prognostic factors from previous statistics, a univariate analysis was performed to screen out the most correlated factors (*P* < 0.05). Afterward, the prognostic factors selected by the univariate analysis were subjected to multivariate Cox proportional hazard regression models for comparisons and to filter out the significant factors (*P* < 0.05). From the direct Kaplan–Meier survival curves drawn from the outcomes of the log-rank test, we compared the ratio of intravesical recurrence-free survival among all the patients and visually analyzed the survival time of certain cases. The aforementioned procedures were performed in SPSS version 13.0 (SPSS Inc., Chicago, USA). To identify the independent risk factors for moderate/severe preoperative urine cytology, a nomogram plot was programmed with R software (R Foundation for Statistical Computing, Vienna, Austria). Receiver operating characteristic (ROC) curves were plotted to compare the ability to estimate for patient mortality between the nomogram and the criteria of the UUT-UC score set by the American Joint Commission on Cancer (AJCC). In addition, calibration plots were also drawn in order to evaluate the potential clinical value of the nomogram. The concordance index (C-index) was calculated by the means of bootstrapping to estimate the probability that the nomogram prognostic models are consistent with the actual observed results. A *P*-value less than 0.05 indicated statistical significance.

### Meta-Analysis

#### Search Strategy

Up to August 2021, we searched the PubMed, Medline, Embase, Cochrane Library, and Scopus databases with the following retrieved words and medical subject headings, including all spellings (“Urine cytology,” “Upper urinary tract,” “Renal pelvis,” “Ureter,” “Urothelial cancer,” “Urothelial carcinoma,” “Bladder cancer,” and “Prognosis,” “Recurrence,” “Intravesical,” and “High grade,” “Muscle invasion,” “Lymphovascular invasion”). Additionally, we manually searched further eligible bibliographies and company reports to ensure the comprehensiveness of the experimental data from the literature. The publication country and time were not restricted.

#### Selection Criteria

The following criteria were used to measure whether valuable literature was collected. The inclusion criteria were as follows: (1) upper urothelial carcinoma was the histologic type, (2) selected samples had corresponding data on the preoperative urine cytology, and (3) relationship was analyzed between preoperative urine cytology and IV-RFS, tumor grade, pathological stages, and lymphovascular invasion. The exclusion criteria were as follows: (1) studies that lacked original data, including reviews, letters to the editor, commentaries, and case reports; (2) studies containing data from earlier studies; (3) articles written by the same authors as another chosen article; (4) studies without original data or no record of intravesical recurrence-free survival, tumor grade, pathological stages, or lymphovascular invasion; and (5) studies with invalid data for calculating the hazard ratio (HR) and its standard error.

#### Data Extraction

According to the aforementioned criteria, two investigators (BF and YuH) undertook a standard process to improve reliability and minimize bias. A third reviewer (YW) examined the extracted results and eliminated any discrepancies between the independent search results. If a study appeared appropriate, then the full text was reviewed. If the relevant studies met the inclusion criteria, then they were included in our meta-analysis. We simultaneously recorded the primary information of eligible articles, including the author, publication year, region, study type, recruitment period, number of patients, baseline characteristics (mean age, sex, etc.), evaluation of positive urine cytology, etc.

#### Heterogeneity Evaluation

The *I* ^2^ and *Q* test were used to quantitatively delineate heterogeneity and measure the percentage of volatility. If the heterogeneity *P*-value was <0.05, a random-effects model rather than the fixed-effect model was employed. If *I* ^2^ was >50%, then heterogeneity was observed among the studies.

#### Sensitivity Analysis

We further adopted a leave-one-out approach to remove individual studies sequentially. If the *P*-value tested by the *Q*-test was greater than 0.05, the fixed-effects model was used after removing the heterogeneous studies. A random-effects model was used if heterogeneity was detected after removing any of the studies.

#### Statistical Analysis

The dichotomous outcomes were compared by assessment of hazard ratios (HRs)/risk ratios (RRs) with 95% CIs. The HRs and 95% CIs were used to demonstrate the statistical outcomes in the prognosis group, while the RRs and 95% CIs were applied in groups of malignant tumor biological behaviors. Overall and subgroup meta-analyses by Begg’s test and Egger’s test were visually reflected by forest and funnel plots to represent the estimated pooled effects to assess the publication bias. In the forest plots, if the diamond crossed the vertical median line, statistical significance was confirmed. Meta-analyses and forest plots were performed using Stata 12.0 (Stata Corporation, College Station, TX, USA).

#### Quality Assessment

The Grading of Recommendations Assessment, Development, and Evaluation (GRADE) approach was used to assess the confidence of evidence for each outcome. Two independent researchers (BF and YuH) downgraded the studies which had five limitations, including risk of bias, inconsistency, indirectness, imprecision, and publication bias (31, 32). Overall quality was classified as “very low,” “low,” “moderate,” or “high” based on the overall judgment. The assessment was performed in using GRADE Pro version 3.6 software.

## Results

### Cohort Study

#### Relationship Between the Examination of Urine Cytology and thr Clinicpathological Characteristics of UUT-UC Patients

**Figure 1:**
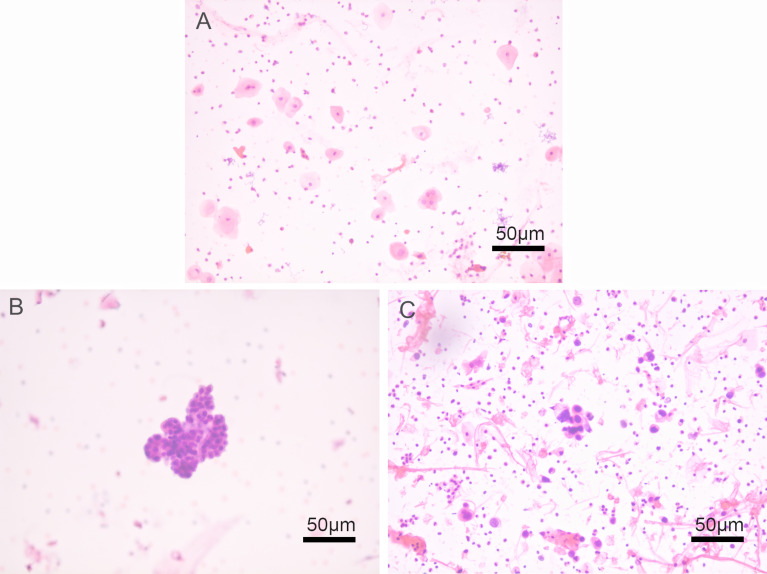
Histological patterns of preoperative urine cytology by HE staining in upper urinary tract urothelial carcinoma (UUT-UC) patients. **(A)** Negative urine cytology at a magnification of ×200; **(B)** suspicious urine cytology at a magnification of ×200; and **(C)** positive urine cytology at a magnification of ×200.

The malignant grades of preoperative urine cytology were detected by HE staining, as shown in **Figure 1**. According to the outcomes of preoperative urine cytology, 231 patients who met our inclusion criteria were divided into a negative group and a positive group. To ascertain the clinical relationship between the outcomes of urine cytology and intravesical recurrence after radical nephroureterectomy in UUT-UC patients, we conducted the training (*n* = 142) and validation cohorts (*n* = 89). The main clinicopathological characteristics in the training and validation cohorts are listed in **Tables 1** and **2**, respectively. The related descriptive clinical and pathological factors included gender, age, smoking, alcohol use, family history of bladder cancer, tumor side, tumor location, tumor focality, pathological stage, histological grade, lymph node status, distant metastasis, and type of surgery. Preoperative positive urine cytology was reported in 68 (47.9%) of 142 patients in the training cohort and 37 (41.6%) of 89 patients in the validation cohort. The correlation between preoperative urine cytology and clinicopathological characteristics was then investigated. We discovered that pathological stage was the only variable positively correlated with preoperative positive urine cytology (*P* = 0.001) in the training cohort, and the association between smoking and preoperative urine cytology (*P* = 0.047) was found in the validation cohort.

**Table 1:** Demographics and clinicopathologic characteristics of 142 patients treated with RNU for UUT-UC in training cohort.

	Preoperative urine cytology		Total	*P* Value
	Negative group	Positive group		
Gender				0.536
Male	43	36	79	
Female	31	32	63	
Age, years				0.064
Less than 69	42	28	70	
69 or Greater	32	40	72	
Smoking				0.405
Non-smoker	48	49	97	
Current smoker	17	15	32	
Former smoker	9	4	13	
Alcohol use				
Non-drinker	51	43	94	0.484
Current drinker	16	14	30	
Former drinker	7	11	18	
Family history of bladder cancer				0.719
No	71	66	137	
Yes	3	2	5	
Tumor side				0.258
Right	44	34	78	
Left	30	34	64	
Tumor location				0.511
Calix or pelvis	40	41	81	
Ureter	29	25	54	
More than 1	5	2	7	
Tumor focality				0.848
Unifocal	65	59	124	
Mutifocal	9	9	18	
Pathologic stage				**0.001**
T_is_-T_1_	43	20	63	
T_2_-T_4_	31	48	79	
Histological grade				0.850
Low	25	24	49	
High	49	44	93	
Lymph node status				0.159
N_o_	71	60	131	
N_1_	3	6	9	
N_x_	0	2	2	
Distant metastasis				0.172
M_0_	72	68	140	
M_1_	2	0	2	
Type of surgery				0.710
Open	49	43	92	
Laparoscopic	25	25	50	

The bold values were applied to highlight P-values which had statistically significance (i.e. P < 0.05).

**Table 2:** Demographics and clinicopathologic characteristics of 89 patients treated with RNU for UUT-UC in validation cohort.

	Preoperative urine cytology		Total	*P* Value
	Negative group	Positive group		
Gender				0.873
Male	29	20	49	
Female	23	17	40	
Age, years				0.926
Less than 69	23	16	39	
69 or Greater	29	21	50	
Smoking				**0.047**
Non-smoker	31	31	62	
Current smoker	16	4	20	
Former smoker	5	2	7	
Alcohol use				
Non-drinker	39	28	67	0.833
Current drinker	7	6	13	
Former drinker	6	3	9	
Family history of bladder cancer				0.768
No	50	36	86	
Yes	2	1	3	
Tumor side				0.599
Right	28	22	50	
Left	24	15	39	
Tumor location				0.910
Calix or pelvis	23	16	39	
Ureter	26	18	44	
More than 1	3	3	6	
Tumor focality				0.851
Unifocal	37	27	64	
Mutifocal	15	10	25	
Pathologic stage				0.796
T_is_-T_1_	11	7	18	
T_2_-T_4_	41	30	71	
Histological grade				0.567
Low	17	10	27	
High	35	27	62	
Lymph node status				0.079
N_o_	47	36	83	
N_1_	5	0	5	
N_x_	0	1	1	
Distant metastasis				0.090
M_0_	52	35	87	
M_1_	0	2	2	
Type of surgery				0.327
Open	12	12	24	
Laparoscopic	40	25	65	

The bold values were applied to highlight P-values which had statistically significance (i.e. P < 0.05).

#### Predictors and Intravesical Recurrence-Free Survival in UUT-UC Patients

**Figure 2:**
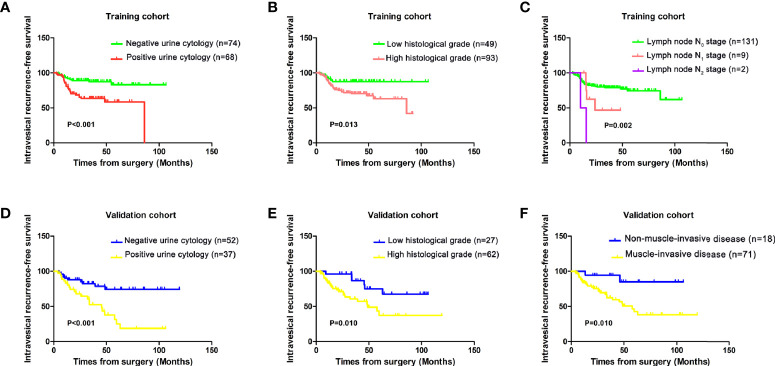
Kaplan–Meier analysis of intravesical recurrence-free survival by **(A)** preoperative urine cytology, **(B)** histological grade, and **(C)** lymph node stage in the training cohort and **(D)** preoperative urine cytology, **(E)** histological grade, and **(F)** muscle invasion in the validation cohort.

The Kaplan–Meier method was applied to determine the relationships between different prognostic factors and recurrence in the bladder. The survival curves showed that preoperative positive urine cytology (*P* < 0.001), higher histological grade (*P* = 0.013), and positive lymph node status (*P* = 0.002) were associated with poor IV-RFS in the training cohort (**Figures 2A–C**). The patients with preoperative positive urine cytology (*P* < 0.001), higher histological grade (*P* = 0.010), and muscle invasion (*P* = 0.010) had lower intravesical recurrence-free survival in the validation cohort (**Figures 2D–F**).

The outcomes of the univariate and multivariate analyses in the training cohort are shown in **Table 3**. After performing univariate and multivariate Cox regression analyses, preoperative urine cytology (HR 3.283, 95% CI 1.558, 6.920, *P* = 0.002) and histological grade (HR 2.683, 95% CI 1.107, 6.502, *P* = 0.029) were detected as independent predictors correlated with intravesical recurrence in the training cohort. Similarly, **Table 4** demonstrates the results of the univariate and multivariate analyses in the validation cohort. Preoperative positive urine cytology (HR 2.975, 95% CI 1.352, 6.548, *P* = 0.007), higher pathologic stage (HR 5.019, 95% CI 1.180, 21.349, *P* = 0.029), and higher histological grade (HR 2.750, 95% CI 1.087, 6.954, *P* = 0.033) also had the potential to predict poor IV-RFS in the validation cohort.

**Table 3:** Univariate and multivariable Cox regression models predicting intravesical recurrence-free survival in 142 patients treated with RNU for UUT-UC in training cohort.

Characteristic	Univariate		Multivariate	
	HR	*P* Value	HR	*P* Value
Gender	0.616 (0.317–1.194)	0.151		
Age	1.748 (0.890–3.435)	0.105		
Family history of bladder cancer	0.046 (0.001–54.700)	0.393		
Smoking	1.450 (0.935–2.247)	0.097		
Alcohol use	1.338 (0.879–2.037)	0.175		
Preoperative urine cytology	3.577 (1.711-7.477)	**0.001**	3.283 (1.558-6.920)	**0.002**
Tumor laterality	1.292 (0.672–2.486)	0.443		
Tumor location	0.954 (0.556–1.638)	0.865		
Tumor focality	0.996 (0.386–2.570)	0.993		
Pathologic stage	1.140 (0.582–2.234)	0.702		
Histological grade	2.897 (1.204–6.971)	**0.018**	2.683 (1.107-6.502)	**0.029**
Lymph node status	2.672 (1.404–5.086)	**0.003**	1.823 (0.947-3.509)	0.072
Distant metastasis	2.464 (0.335–18.132)	0.376		
Type of surgery	0.848 (0.417–1.727)	0.650		

The bold values were applied to highlight P-values which had statistically significance (i.e. P < 0.05).

**Table 4:** Univariate and multivariable Cox regression models predicting intravesical recurrence-free survival in 89 patients treated with RNU for UUT-UC in validation cohort.

Characteristic	Univariate		Multivariate	
	HR	*P* Value	HR	*P* Value
Gender	1.327 (0.641–2.744)	0.446		
Age	1.001 (0.490–2.045)	0.997		
Family history of bladder cancer	0.047 (0.001–415.758)	0.510		
Smoking	0.440 (0.203–0.952)	**0.037**	0.519 (0.238-1.130)	0.099
Alcohol use	0.512 (0.253–1.034)	0.062		
Preoperative urine cytology	3.575 (1.678-7.615)	**0.001**	2.975 (1.352-6.548)	**0.007**
Tumor laterality	0.608 (0.291–1.272)	0.186		
Tumor location	0.888 (0.467–1.689)	0.717		
Tumor focality	0.772 (0.314–1.897)	0.572		
Pathologic stage	5.423 (1.291–22.789)	**0.021**	5.019 (1.180-21.349)	**0.029**
Histological grade	3.105 (1.262–7.642)	**0.014**	2.750 (1.087-6.954)	**0.033**
Lymph node status	0.530 (0.083–3.378)	0.502		
Distant metastasis	1.703 (0.229–12.681)	0.603		
Type of surgery	0.570 (0.278–1.167)	0.124		

The bold values were applied to highlight P-values which had statistically significance (i.e. P < 0.05).

#### Nomogram of Prognostic Prediction Based on Preoperative Urine Cytology

**Figure 3:**
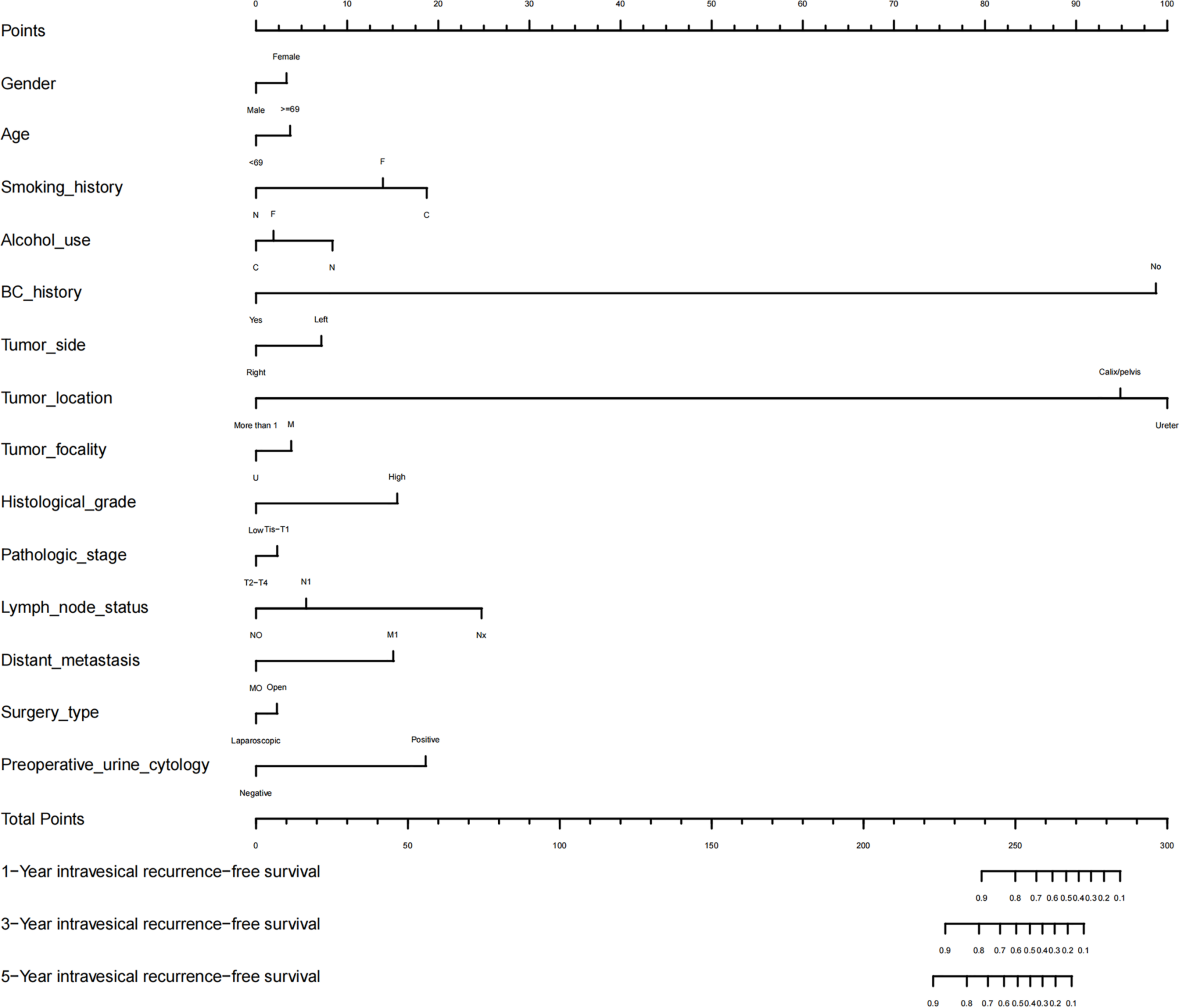
Nomogram for 1-, 3-, and 5-year IV-RFS predictions of UUT-UC and associated predictive performance. The independent prognostic factors (including gender, age, smoking, alcohol use, history of bladder cancer, tumor side, tumor location, tumor focality, histological grade, pathologic stage, lymph node status, distant metastasis, and surgery type and preoperative urine cytology) were combined with the point designations to obtain the total prognostic score. “N” represented non-smoker/drinker, “C” represented current smoker/drinker, “F” represented former smoker/drinker, “U” represented unifocal tumor, “M” represented multifocal tumor, and “BC” represented bladder cancer.

As shown in **Figure 3**, we established a novel nomogram using R language. Gender, age, smoking, alcohol use, history of bladder cancer, tumor side, tumor location, tumor focality, histological grade, pathologic stage, lymph node status, distant metastasis, surgery type, and preoperative urine cytology were combined. According to the constructed nomogram model, the clinician can locate the data on the nomogram, sum the score, and obtain a total prognostic point. The 1-, 3-, and 5-year IV-RFS rates of UUT-UC patients can be predicted accordingly.

**Figure 4:**
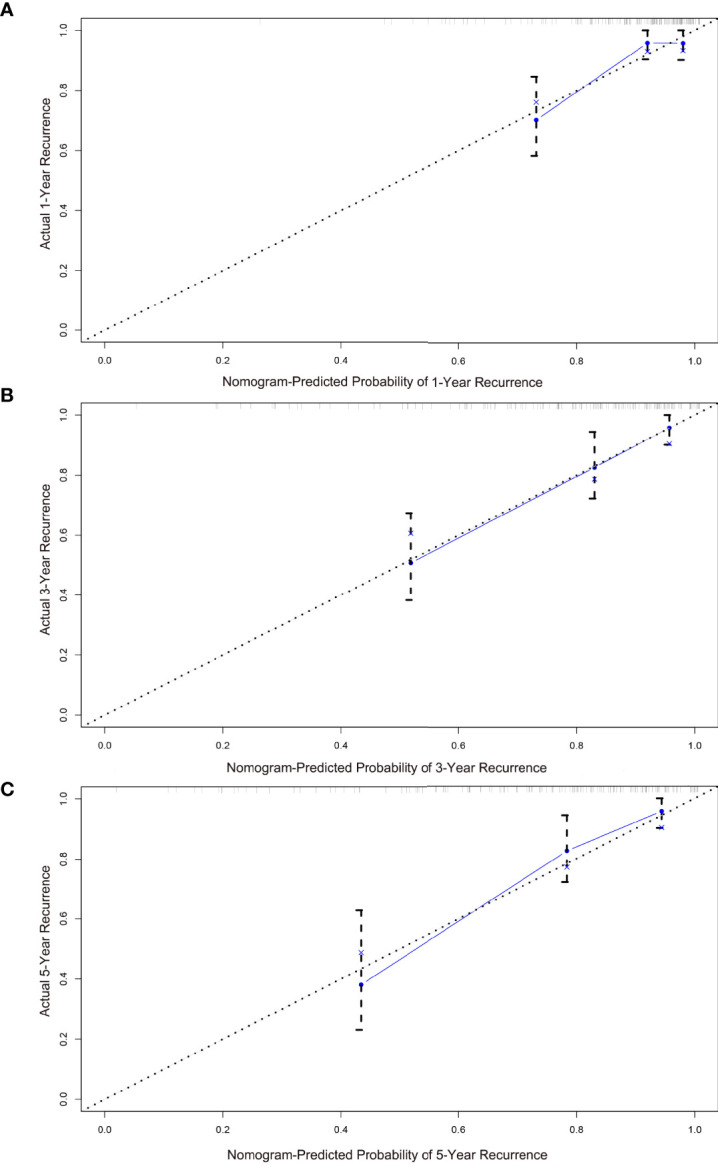
Calibration plots for the nomogram predictions of **(A)** 1-, **(B)** 3-, **(C)** 5-year IV-RFS in the training cohort. The nomogram-predicted probability is plotted on the *x*-axis, and actual recurrence is plotted on the *y*-axis. The gray dotted line represents the ideal curve of the calibration models. The blue broken line demonstrates the actual predictive accuracy, the bootstrap-corrected estimates are visually shown by blue X, and vertical bars represent the 95% CIs.

**Figure 5:**
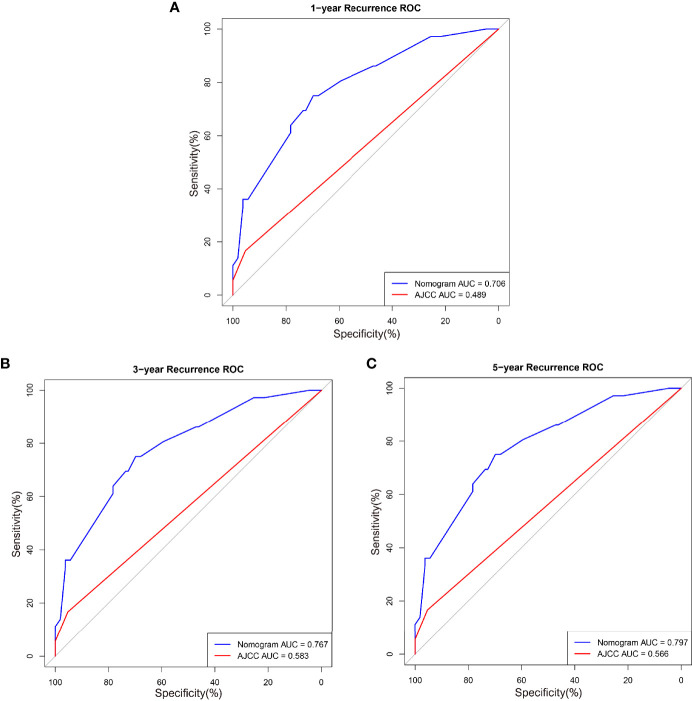
ROC curves comparing the effectiveness of predicting **(A)** 1-, **(B)** 3-, and **(C)** 5-year IV-RFS between the nomogram and the American Joint Commission on Cancer (AJCC) score in the training cohort. Nomogram-predicted intravesical recurrence-free survival rates are indicated by the blue lines, and AJCC-predicted overall survival rates are indicated by the red lines.

The calibration and discrimination ability for internal validation was assessed by calibration curves and the C-index. The calibration plots are shown in **Figures 4A–C**. In comparison with the prediction of 1- and 5-year IV-RFS, we observed that the actual curves were more consistent with the ideal curves in the 3-year nomogram. Compared with AJCC models, the ROC curves of 1-, 3-, and 5-year urine cytology nomogram models revealed better discrimination efficacy, as shown in **Figures 5A–C**. The AUCs of the 1-, 3-, and 5-year AJCC models and the urine cytology nomogram models were equal to 0.489 *vs*. 0.706, 0.583 *vs*. 0.767, and 0.566 *vs*. 0.797, respectively. Good discrimination was also obtained for the bootstrapping method, and the C-index of the nomogram model was equal to 0.673.

**Figure 6:**
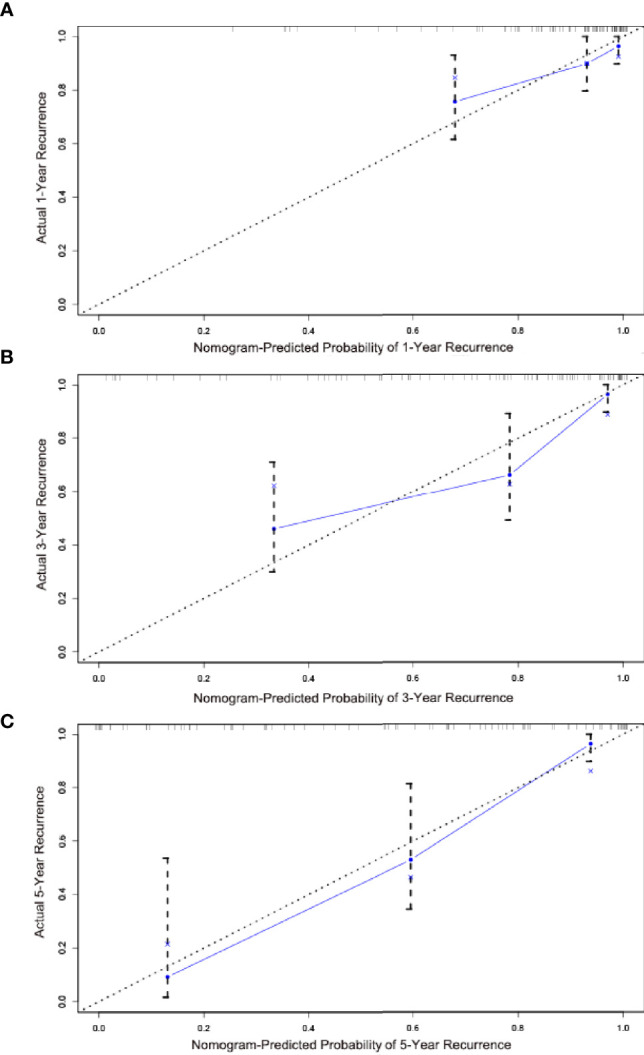
Calibration plots for the nomogram predictions of **(A)** 1-, **(B)** 3- , and **(C)** 5-year IV-RFS in the validation cohort. The nomogram-predicted probability is plotted on the *x*-axis; actual recurrence is plotted on the *y*-axis. The gray dotted line represents the ideal curve of the calibration models. The blue broken line demonstrates the actual predictive accuracy, the bootstrap-corrected estimates are visually shown by blue X, and the vertical bars represent the 95% CIs.

**Figure 7:**
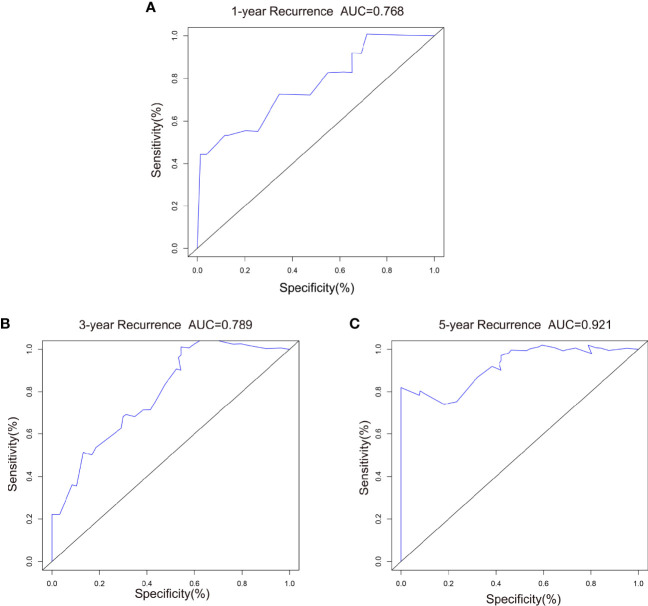
ROC curves comparing the effectiveness of predicting **(A)** 1-, **(B)** 3-, and **(C)** 5-year IV-RFS between the nomogram and the AJCC score in the validation cohort. Nomogram-predicted intravesical recurrence-free survival rates are indicated by the blue lines.

For external validation, we evaluated the nomogram by using an independent validation cohort. Good consistency between the predicted and actual curves was demonstrated on calibration plots of the 3-year nomogram (**Figures 6A–C**). The C-index of the external validation was 0.788, indicating that the nomogram model had good discrimination efficacy for predicting IV-RFS in patients with UUT-UC. Compared with 1- and 3-year ROC curves, better predictive performance was shown on the 5-year ROC curves (**Figures 7A–C**). The 1-, 3-, and 5-year AUC values were equal to 0.768, 0.789, and 0.921, respectively.

In summary, we speculate that the prognostic nomogram model has a great potential to predict 1-, 3-, and 5-year IV-RFS.

### Meta-Analysis

#### Method of Systematic Selection of Relevant Publications

**Figure 8:**
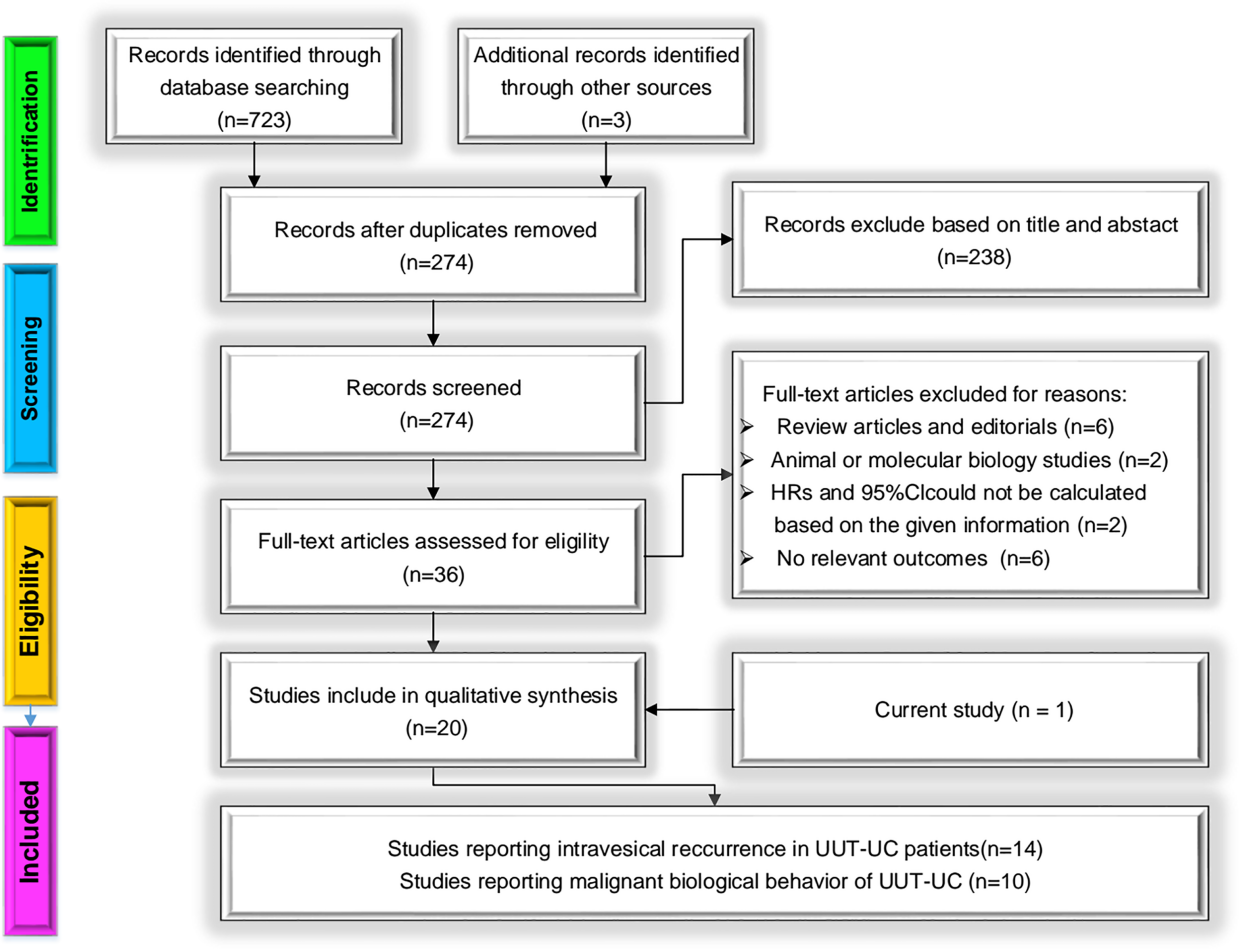
Methodologic flow diagram for the selection of articles.

The detailed process of selection is shown in **Figure 8**. A total of 723 initial studies were identified from electronic databases and literature searches, and additional records were found from other approaches (*n* = 3). After removing duplicated publications (*n* = 274) and excluding articles by title and abstract (*n* = 238), 36 studies remained. With the careful assessment of the remaining studies, 16 studies were excluded for four reasons: 1) review articles and editorials, 2) unavailable HRs and 95% CIs, 3) animal or molecular biology studies, and 4) not relevant outcomes. Twenty candidate studies remained. Adding these to our cohort study, 21 independent studies were finally included in our meta-analysis. There were two available datasets in two articles about prognosis [Kentaro K, 2019 (33); Long X, 2016 (24)]. We accepted different studies from the same group.

#### Meta-Analysis of the Correlation Between Preoperative Positive Urine Cytology and Intravesical Recurrence in UUT-UC Patients

##### The Main Characteristics of Eligible Studies

The major characteristics of 14 studies about intravesical recurrence are summarized in **Table 5**. They were published from 2010 to 2020. One, four, and nine studies assessed patients from Korea (21), China (23, 24, 28), and Japan (30, 33–40), respectively. In addition, one, three, and four studies were RCT (36), case–control (21, 35, 38), and single-arm studies (23, 37, 39, 40), respectively, and the others were retrospective cohort searches (24, 28, 30, 33, 34). The size of the studies varied from 36 to 1,563, with a total number of 6,140 patients. Only one publication did not provide the recruitment period, and demographics were missing from only one publication. Seven studies showed the assessment of positive urine cytology.

**Table 5:** Main characteristics of studies about the intravesical recurrence-free survival included in the meta-analysis.

First author (year)	Country	Study type	Recruitment period	No. of subjects	Demographics (age, sex)	Assessment of positive urine cytology	Reported endpoints
Liu W (2020)	China	Cohort study; Retrospective	2012-2019	315	67.0 M:F=192:123	Defined as a positive result and/or a suspicious report	2.210(1.060-4.640)
Kentaro K (2019)	Japan	Cohort study; Retrospective	1995-2009	1563	70.0 M:F=1914:754	NR	1.210(1.020-1.430)
				1245			1.210(1.000-1.480)
Long X (2016)	China	Cohort study; Retrospective	2004-2012	159	62.0 M:F=113:46	NR	2.173(1.084-4.350)
				161	60.8 M:F=120:41		1.143(0.544-2.400)
Ishioka J (2015)	Japan	Case-control study; Retrospective	1995-2010	754	69.0 M:F=526:228	NR	1.259(0.926-1.711)
Narukawa T (2015)	Japan	Case-control study; Retrospective	1995-2012	133	66.0 M:F=101:32	NR	1.080(0.630-1.850)
Fang D (2014)	China	Single arm study	2000-2010	438	NR M:F=187:251	Indication of malignancy and the presence of atypical cells that were highly suggestive of urothelial carcinoma	1.160(0.810-1.660)
Shibuya T (2014)	Japan	Single arm study	2002-2012	54	NR	NR	2.680(1.110-6.450)
Cho DS (2013)	Korea	Case-control study; Retrospective	1994-2009	78	65.0 M:F=58:20	Class 4 or 5 findings according to the Papanicolaou classification	4.606(1.450-14.633)
Ito A (2013)	Japan	RCT	NR	36	69 M:F=43:29	At least one positive finding among multiple examinations	5.540(1.120-27.500)
Tanaka N (2013)	Japan	Cohort study; Retrospective	1994-2010	474	69.0 M:F=346:128	One that reported suspicious or positive results, or both	1.410(1.080-1.850)
Kobayashi Y (2012)	Japan	Single arm study	2005-2009	288	71.4 M:F=197:91	NR	1.977(1.310-2.983)
Hirano D (2012)	Japan	Cohort study; Retrospective	1995-2010	151	68.0 M:F=121:30	NR	0.948(0.498-1.807)
Takaoka E (2010)	Japan	Single arm study	1989-2007	60	64.7 M:F=40:20	Class 3, 4 or 5 findings according to the Papanicolaou classification	1.560(0.481-5.053)
This study	China	Cohort study; Retrospective	2008-2018	142	67.1 M:F=79:63	One that reported suspicious or positive results, or both	3.283(1.558-6.920)
				89	68.0 M:F=49:40		2.975(1.352-6.548)

RCT, randomized controlled trial; NR, no report; M:F, male-female ratio.

##### Heterogeneity and Sensitivity Analyses

**Figure 9:**
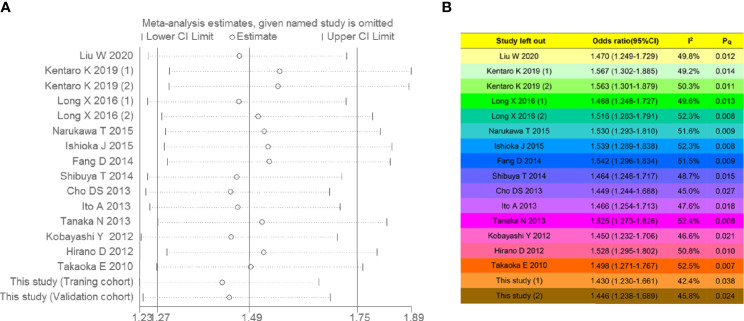
Sensitivity analysis for intravesical recurrence-free survival. **(A)** Forest plot and **(B)** leave-one-study-out sensitivity analysis. If the *P*-values of the *Q*-test (*P_Q_*) were more than 0.05, the individual studies were heterogeneous. Low, moderate, and high heterogeneity corresponded to *I* ^2^ ranges of 0%–30%, 30%–50%, and 50%–100%, respectively.

A random-effects model was selected because moderate heterogeneity was detected (*I* ^2^ = 49.4%, *P* = 0.011). A sequential sensitivity analysis was conducted to evaluate heterogeneity in each study, as shown in **Figure 9A**. By removing any of the individual studies, statistical fluctuations of the combined HRs were not observed, indicating that the pooled data combined with the random-effects model have great reliability. The complete *I* ^2^ statistical values and *P*-values of the *Q*-test in the leave-one-study-out sensitivity analysis are summarized in **Figure 9B**.

##### Outcomes Using Random-Effects Models, Publication Bias, and GRADE

**Figure 10:**
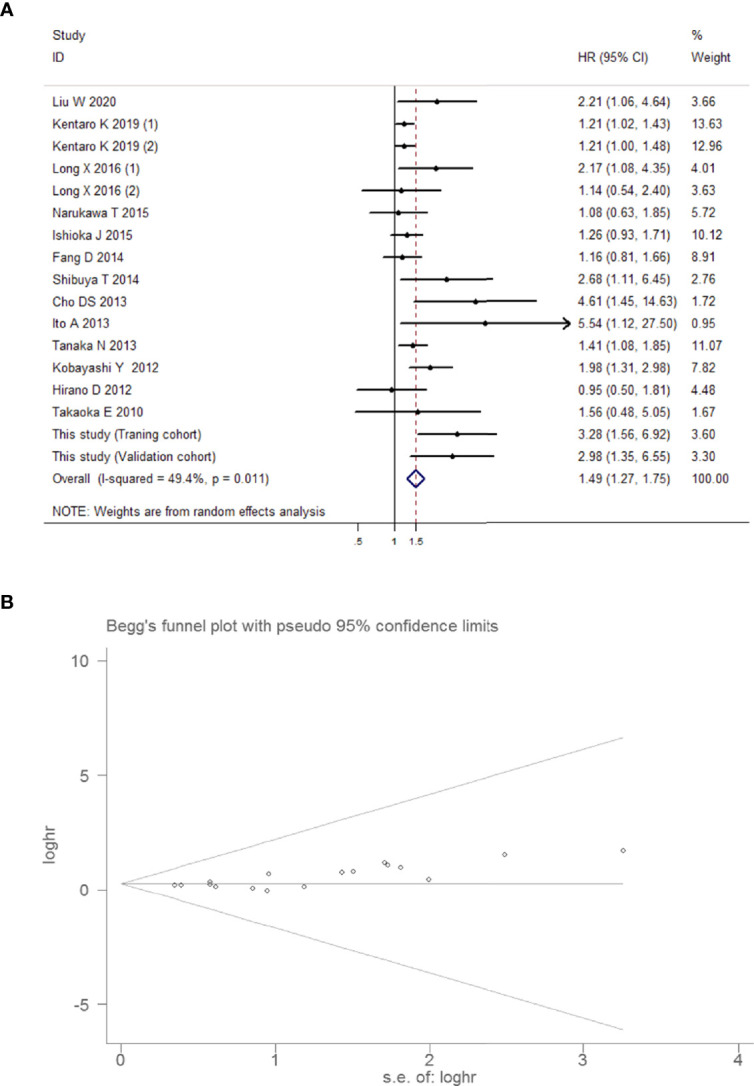
Overall meta-analysis for intravesical recurrence-free survival. **(A)** Forest plot showing intravesical recurrence-free survival in UUT-UC patients with preoperative positive urine cytology in the total analysis. Squares in each trial indicate the hazard ratios, the horizontal line that traverses the square indicates the 95% confidence interval (CI), and diamonds indicate the estimated combined effect. According to the heterogeneity analysis, the Mantel–Haenszel random-effects model was selected. **(B)** Funnel plot for intravesical recurrence-free survival in UUT-UC patients with preoperative positive urine cytology.

Forest plots are depicted in **Figure 10A**, which presents the relative risk estimates from 14 studies for the relationship between preoperative urine cytology and intravesical recurrence. The overall analysis showed that the combined HR was 1.49 (95% CI 1.27, 1.75, *P* < 0.001), suggesting that preoperative positive urine cytology was associated with a 49% increased risk of intravesical recurrence. Visually, the shape of Begg’s funnel plots (**Figure 10B**) revealed evidence of obvious asymmetry (*z*-value of Begg’s test = 0.005). Statistically, the positive result in Egger’s test indicated moderate publication bias (*P* = 0.001). The pooled findings of the GRADE analysis (**Table 6**) achieved a very low quality of evidence supporting poorer intravesical recurrence-free survival with preoperative positive urine cytology due to indirectness (findings being restricted to limited regions) and imprecision (large width of the confidence interval around the pooled hazard ratios).

**Table 6:** The overall quality of evidence in pooled findings from eligible studies^a^.

Outcomes	No. of studies	Design	Certain quality assessment					Summary of findings	
			Risk of bias	Inconsistency	Indirectness	Imprecision	Other considerations	Relative effect (95% CI)	Quality
Intravesical recurrence-free survival	17	Observational studies	Not serious	Not serious	Serious^c^	Serious^e^	None	HR 1.49 (1.27 to 1.75)	⊕⊝⊝⊝ VERY LOW
Risk of high-grade UUT-UC	10	Observational studies	Not serious	Serious^b^	Not serious	Serious^e^	None	RR 1.23 (1.00 to 1.51)	⊕⊝⊝⊝ VERY LOW
Risk of muscle-invasive UUT-UC	8	Observational studies	Not serious	Serious^b^	Not serious	Serious^e^	None	RR 1.16 (0.98 to 1.37)	⊕⊝⊝⊝ VERY LOW
Risk of lymphovascular-invasive UUT-UC	3	Observational studies	Not serious	Not serious	Serious^d^	Very serious^e^	None	RR 1.37 (0.81 to 2.31)	⊝⊝⊝⊝ VERY LOW

^a^The quality assessment was based on GRADE approach; ^b^High levels of heterogeneity in pooled findings; ^c^Results restricted to China/Japan/Korea; ^d^Limited findings from China/Japan; ^e^The range of true values in each pooled studies was too large in varying degrees.

##### Subgroup Analysis

**Figure 11:**
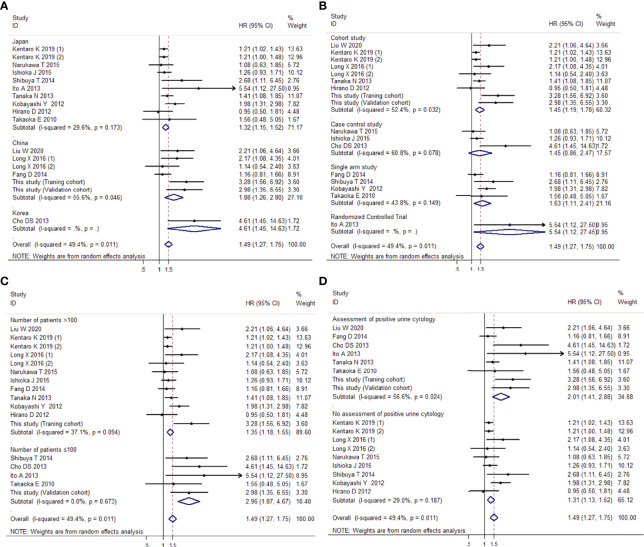
Forest plots showing the risk of intravesical recurrence in UUT-UC patients with preoperative positive urine cytology by subgroup analysis of the **(A)** region, **(B)** study types, **(C)** sample size, and **(D)** assessment of urine cytology. Squares in each trial indicate the hazard ratios, the horizontal line that traverses the square indicates the 95% confidence interval (CI), and diamonds indicate the estimated combined effect. According to the heterogeneity analysis, the Mantel–Haenszel random-effects model was selected.

In the subgroup analysis by region, a significant difference was noted between the Japan subgroup (HR 1.32, 95% CI 1.15, 1.52, *P* < 0.001), the China subgroup (HR 1.88, 95% CI 1.26, 2.80, *P* = 0.002), and the Korea subgroup (HR 4.61, 95% CI 1.45, 14.63, *P* = 0.01). Under the analysis of study types, precise evidence was obtained showing that preoperative positive urine cytology was related to an increase in intravesical recurrence, not only in the subgroup of the cohort studies (HR 1.45, 95% CI 1.19, 1.78, *P* < 0.001) but also in the subgroup of the single-arm study (HR 1.63, 95% CI 1.11, 2.41, *P* = 0.013) and RCT study (HR 5.54, 95% CI 1.12, 27.45, *P* = 0.036). In the subgroup analysis by sample size, when the sample size was more than 100, the risk of intravesical recurrence was 1.35 times higher in patients with positive urine cytology than in those with negative cytology (95% CI 1.18, 1.55, *P* < 0.001). When the sample size was 100 or less, the same trend was seen (HR 2.96, 95% CI 1.87, 4.67, *P* < 0.001). In addition, we explored whether the assessment of urine cytology influenced the preoperative urine cytology and intravesical recurrence. The results showed that preoperative positive urine cytology was associated with an increase in intravesical recurrence, not only in the group with the assessment of urine cytology (HR 2.01, 95% CI 1.41, 2.88, *P* < 0.001) but also in the group without assessment of urine cytology (HR 1.31, 95% CI 1.13, 1.52, *P* < 0.001). **Figures 11A–D** show all the forest plots.

#### Meta-Analysis of the Correlation Between Preoperative Positive Urine Cytology and Tumor Malignant Biological Behavior for UUT-UC

##### Article Descriptions

**Table 7** outlines the main characteristics of 10 studies about tumor malignant biological behavior. These studies contained 2,463 samples. Considering the selected articles from 2007 to 2021, four studies were retrospective cohort studies (28, 30, 41), and the remaining were case–control studies (29, 42–46). There were four studies from China (28, 42, 45), two studies from America (29, 41), one study from Germany (43), two studies from Japan (30, 46), and one study from Spain (44). In these studies, nine of them involved the risk of high-grade UUT-UC (28–30, 41–45). The risks of muscle-invasive UUT-UC and lymphovascular-invasive UUT-UC emerged in seven studies (30, 41–45) and three studies (28, 30, 46), respectively.

**Table 7:** Main characteristics of studies about tumor malignant biological behaviors included in the meta-analysis.

First author (year)	Country	Study type	Recruitment period	No. of subjects	Demographics (age, sex)	Assessment of positive urine cytology	Reported endpoints
Kuroda K (2021)	Japan	Case-control study; Retrospective	NR	145	70.0 M:F=109:36	NR	LVI: 3.439(1.373-8.613)
Liu W (2020)	China	Cohort study; Retrospective	2012-2019	315	67.0 M:F=192:123	Defined as a positive result and/or a suspicious report	HG: 1.190(1.060-1.350) LVI: 0.860(0.560-1.320)
Maruschke M (2017)	Germany	Case-control study; Retrospective	1996-2011	113	NR	NR	HG: 1.010(0.450-2.250) MI: 0.890(0.490-1.590)
Wang L (2014)	America	Case-control study; Retrospective	2000-2011	65	69.8 M:F=36:29	One that reported suspicious or positive results, or atypical diagnosis	HG: 1.870(1.240-2.840)
Chen X (2013)	China	Case-control study; Retrospective	2002-2010	693	NR M:F=307:386	Defined as a positive result and/or a suspicious report	HG: 1.365(0.938-1.987) MI: 1.519(1.043-2.212)
Tanaka N (2013)	Japan	Cohort study; Retrospective	1994-2010	474	69.0 M:F=346:128	One that reported suspicious or positive results, or both	HG: 1.200(1.040-1.380) MI: 1.110(0.980-1.260) LVI: 1.330(1.050-1.680)
Messer J (2011)	America	Cohort study; Retrospective	1997-2008	326	70.0 M:F=202:124	Presence of malignant cells	HG: 0.640(0.530-0.770) MI: 0.820(0.610-1.110)
Xu C (2010)	China	Case-control study; Retrospective	NR	71	64.8 M:F=54:17	NR	HG: 2.330(1.330-4.070) MI: 1.550(0.820-2.930)
Marín-Aguilera M (2007)	Spain	Case-control study; Retrospective	2003-2006	30	66.0 M:F=25:5	One that reported suspicious or positive results, or both	HG: 2.310(1.350-3.960) MI: 1.170(0.600-2.270)
This study	China	Cohort study; Retrospective	2008-2018	142	67.1 M:F=79:63	One that reported suspicious or positive results, or both	HG: 0.980(0.770-1.240) MI: 1.690(1.210-2.300)
				89	68.0 M:F=49:40		HG: 1.080(0.830-1.420) MI: 1.030(0.830-1.270)

LVI, lymphovascular invasion; HG, high grade; MI, muscle invasion; NR, no report; M:F, male-female ratio.

##### Meta-Analysis of the Correlation Between Preoperative Positive Urine Cytology and the Risk of High-Grade UUT-UC

**Figure 12:**
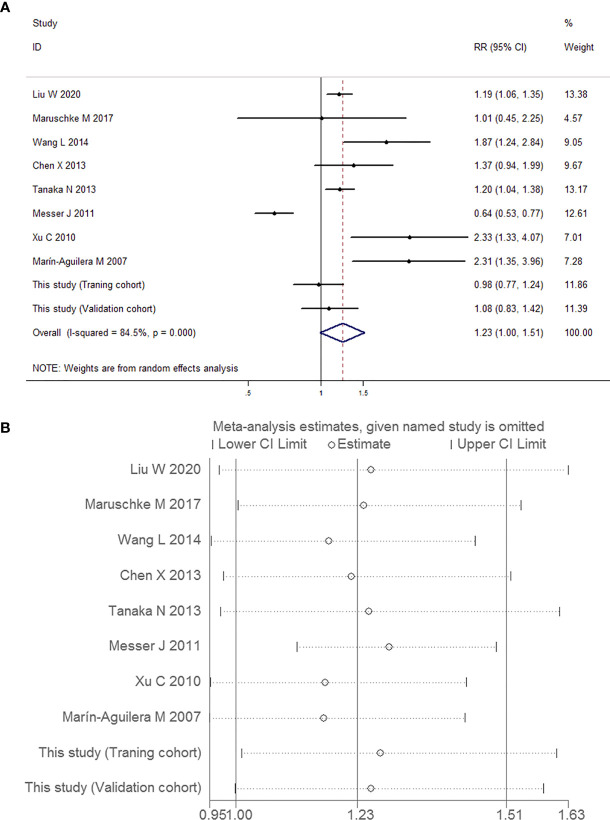
Forest plots for the risk of high-grade UUT-UC in patients with preoperative positive urine cytology. **(A)** Meta-analysis. **(B)** Sensitivity analysis.

A total of 2,318 patients were pooled in this analysis. As shown in **Figures 12A, B**, there was substantial heterogeneity overall, indicating that a random-effects model analysis was applied (*I* ^2^ = 84.5%, *P* < 0.001). No significant risk of high-grade tumor was observed in the UUT-UC patients with preoperative positive urine cytology (RR 1.23, 95% CI 1.00, 1.51, *P* = 0.055). Due to the high heterogeneity and wide confidence interval, the pooled findings achieved a very low quality of evidence due to their inconsistency and imprecision (**Table 6**).

##### Meta-Analysis of the Correlation Between Preoperative Positive Urine Cytology and the Risk of Muscle-Invasive UUT-UC

**Figure 13:**
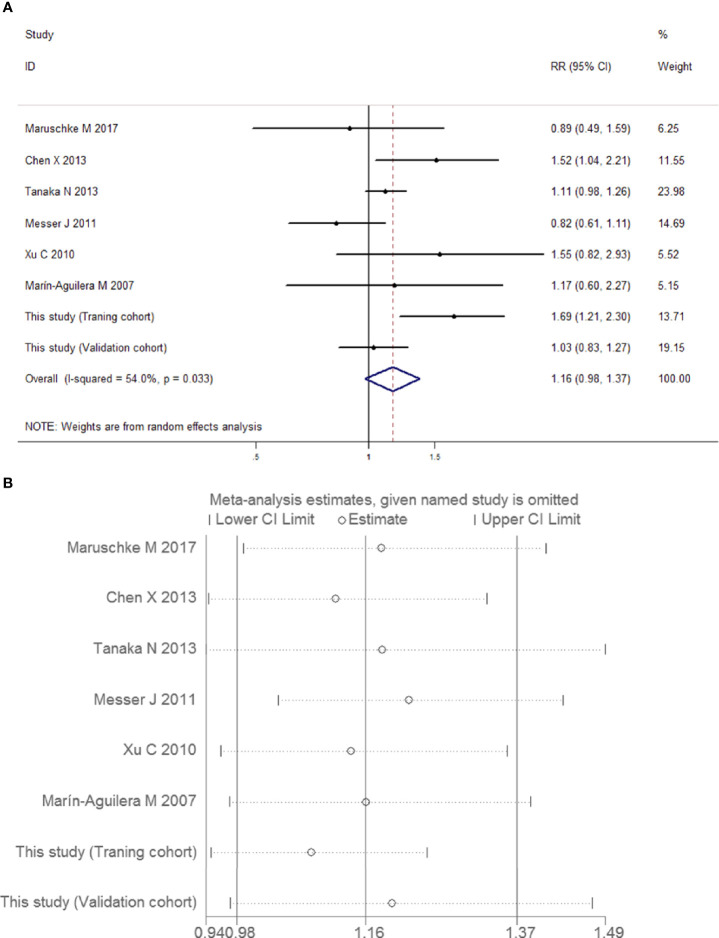
Forest plots for the risk of muscle-invasive UUT-UC in patients with preoperative positive urine cytology. **(A)** Meta-analysis. **(B)** Sensitivity analysis.

Similarly, the outcomes of relationship and heterogeneity are demonstrated in **Figures 13A, B**. A total of 1,938 patients with muscle-invasive UUT-UC were enrolled. With severe heterogeneity detected among these studies (*I* ^2^ = 54.0%, *P* = 0.033), the combined analysis using a random-effects model demonstrated that preoperative urine cytology had no obvious association with the risk of muscle-invasive UUT-UC (RR 1.16, 95% CI 0.98, 1.37, *P* = 0.085). Once again, the quality of evidence regarding muscle-invasive UUT-UC was very low (**Table 6**).

##### Meta-Analysis of the Correlation Between Preoperative Positive Urine Cytology and the Risk of Lymphovascular-Invasive UUT-UC

**Figure 14:**
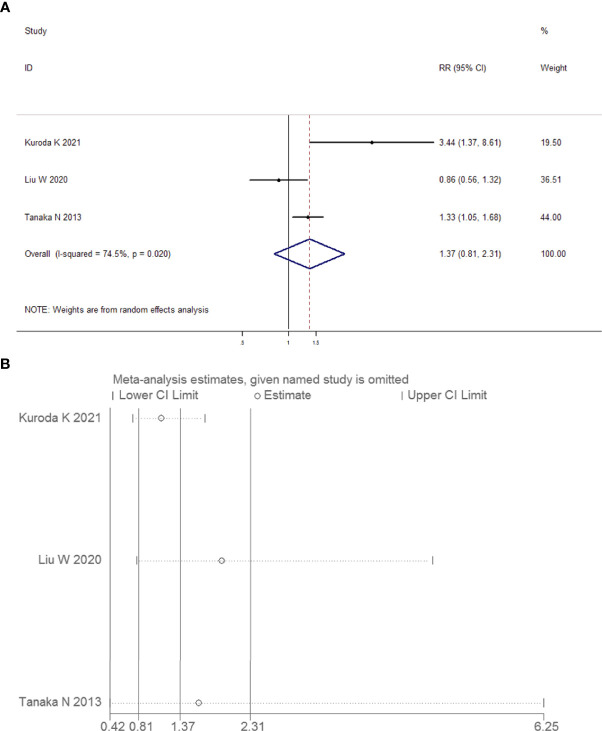
Forest plots for the risk of lymphovascular-invasive UUT-UC in patients with preoperative positive urine cytology. **(A)** Meta-analysis. **(B)** Sensitivity analysis.

We combined the data of 934 patients from two eligible articles and conducted the meta-analysis. The forest plot and the sensitivity test are depicted in **Figures 14A, B**, respectively. A random-effects model analysis was applied because evidence of heterogeneity was observed (*I* ^2^ = 74.5%, *P* = 0.020). The pooled RR was equal to 1.37, but it was not statistically significant (95% CI 0.81, 2.31, *P* = 0.245). The results showed that the preoperative positive urine cytology was not related to the risk of lymphovascular invasion in UUT-UC. The limitation of the findings to China or Japan and the large ranges of true values in two eligible articles indicated that the evidence of outcomes had a very low quality (**Table 6**).

## Discussion

In most studies, the ratio of intravesical recurrence has ranged from 22% to 50% in patients with UUT-UC who underwent RNU (47). Recent studies about the association between preoperative positive urine cytology and intravesical recurrence in patients with UUT-UC in northeast China were relatively rare and controversial. Inspired by the above, we collected data from three clinical centers and conducted a retrospective cohort study. In our Kaplan–Meier survival and univariate and multivariate Cox regression analyses, we found that preoperative urine cytology was a significant factor for bladder cancer recurrence in the training cohort. The use of the validation cohort verified the reliability of the outcomes from the training cohort. Meanwhile, we conducted a nomogram model to further determine the predictive value of preoperative urine cytology. Actually, as various risk factors correlated with intravesical recurrence have been found and analyzed, studies differ significantly in the identification of multifactorial prognosis. For patients with UUT-UC and recurrence of bladder cancer, tumor location in the ureter or tumor multifocality has been validated as prognostic factors in the recurrence of non-muscle-invasive bladder cancer (48–50). The incidence of multiple upper urothelial carcinomas was significantly higher than that of single urothelial carcinomas in patients with urothelial tumor recurrence (51). In addition, tumor size, preoperative hydronephrosis, and ureterorenoscopy are more likely to lead to postoperative recurrence in the bladder (52–54). Some of those conclusions are consistent with ours. The differences among studies explain the diversity of characteristics of urothelial carcinoma, and they have led to uncertainties in clinical follow-up and treatment, which should be studied further in long-term, systematic, large-sample research.

In our meta-analysis, preoperative positive urine cytology was associated with a 49% increased risk of intravesical recurrence. Interestingly, there is an attractive hypothesis that can explain the mechanism of urinary abscission cytology in the prediction of recurrence in the bladder after upper urothelial neoplasms. In a previous study, the dissemination and floating of cancer cells from UUT-UC in the bladder occurred postoperatively (55, 56). During radical nephroureterectomy, the bladder is cut open, and a urethral catheter is placed, resulting in an increased degree of bladder exposure. The injured urothelium is more likely to provide a site for adherence compared with complete urothelium. Along with continuously monitoring the removal of postoperative remnants, urinary bladder suspensions can be used to determine whether tumor cells have adhered to the damaged urothelium and proliferated (57, 58).

In fact, tumors can occur in one or more sites simultaneously or at different times. Recurrent bladder cancer after upper urinary tract urothelial carcinoma is a kind of multiple-organ carcinoma of the urinary tract epithelium (59, 60). It is universally acknowledged that the multicentricity of lesions plays an essential role in the biological characteristics of urothelial carcinoma (55, 58, 59). Based on our findings and implantation theory, it seems that urothelial multiorgan carcinoma originates from the same cell clone. According to this view, urothelial carcinoma originates from a single cell clone, and urothelial multiorgan carcinoma is caused by tumor cells implanting along the direction of urine flow and migrating intraepithelially to form multiple tumor lesions (55, 61). Many conditions can be explained by the implantation theory, such as unilateral upper urinary tract urothelial carcinoma, tumors formed in the same direction as urine flow, and consistent tumor histologic patterns in multiple organs. Although both UUT-UC and bladder cancer originate from similar transitional epithelium of the urinary tract, next-generation sequencing may provide insights about differences in genetic landscapes between UUT-UC and bladder cancer. In northern China, Yang et al. reported that the distribution of driver genes is significantly different between UUT-UC with PIK3CA, TP53, and FGFR3 mutations and bladder cancer with BRCA1 mutations. Meanwhile, patients with bladder cancer had higher levels of PD-L1 than those with UUT-UC (62). In eastern China, the mutation frequencies of GPR126 intron 6, TERT, and PLEKHS1 promoters in UUT-UC patients were significantly lower than those in patients with bladder cancer (63). The U.S. research team of Petros et al. showed that patients who develop UUT-UC after bladder cancer may predominantly show a more basal-like subtype, while synchronous and primary UUT-UC patients appear to have a luminal-like subtype, which results in a different distribution of fibroblast and immune cell gene expression (64).

From the perspective of tumorigenesis, another theory is the multifocal origin theory. The multicentricity of urothelial carcinoma is caused by chromosome deletion and gene mutation of normal cells through the impact of local carcinogenic factors, thus forming independent cell clones. These clones proliferate to produce multiple tumor lesions at the same or different times. This theory can explain the bilateral upper urinary tract tumors appearing at the same or different times and the long-term neoplasms that occur in the same direction. Some scholars still emphasize that intravesical recurrence and even multiorgan carcinoma in the reverse direction from urinary flow are related to tumor cell implantation. In fact, studies have reported that multiple or recurrent urothelial carcinomas likely arise from the same tumor cell implantation rather than from multicentric tumors (55, 59). In recent years, genomic profiles were applied to investigate tumor genomic characterization between primary UUT-UC and subsequent bladder cancer by van Doeveren et al., who found that UUT-UC tissues and paired bladder cancer had clonal relationships by analysis of shared genomic variants (65). Audenet et al. reported that UUT-UC patients with genetic mutations, including FGFR3, CCND1, KDM6A, and TP53, had a higher risk of subsequent bladder recurrence after UUT-UC (1). Above all, whether tumor cell implantation causes recurrent bladder cancer after urothelial carcinoma is worthy of further study for germline and somatic gene alterations (60, 66).

Although we performed a cohort study and meta-analysis of the data in the published literature to overcome the limitation of our single-center sample source to some extent and further confirm the accuracy of our research results further, our study has some potential limitations. First, our limited samples were enrolled from three medical centers, which may restrict the universality of our retrospective findings and cause certain limitations in the statistical analysis of predictors. Meanwhile, because our nomogram model was limited by confined samples, further enlargements on data collection and integration of other prognostic factors will refine reliability and availability for clinical prediction. It is necessary to conduct additional work at more clinical centers with larger included samples to facilitate objective and accurate findings, which will contribute to the impact of preoperative positive urine cytology on the prognostic assessment of UUT-UC patients. Moreover, the meta-analysis results are also problematic. Although we searched for literature from multiple sources and even included unpublished trials and abstracts, which can increase the risk of null results, we could not eliminate potential bias. Publication bias might have been caused by several factors: 1) in these articles, we selected the hazard ratios from the multivariate survival analysis. Univariate data will be adopted when the available results emerge. 2) If the HRs and their variance were not reported, we performed a calculation from the survival comparison statistics and its variance, which has less reliability than direct data (67–69). In addition, different diagnostic criteria for urinary cytology are used among different countries and regions, and the quality of this study would have been improved by standardized protocols. Furthermore, the number of articles about malignant tumor biological behavior was limited. The included samples could have led to heterogeneity among the groups, which would lead to an inconspicuous association between preoperative urine cytology and malignant tumor biological behavior risk. We expect that more similar studies could be included by other researchers to reach a reasonable conclusion.

## Conclusion and Outlook

With the development of molecular biology approaches, the processes of urothelial carcinoma pathogenesis have been widely and deeply explored. We classified bladder cancer by gene sequencing and comprehensive profiling of RNA expression and used DNA sequencing results from biopsy to understand survival distributions and prognostic factors (70–72). This new understanding of heterogeneous diseases, such as UUT-UC and bladder cancer, can improve outcomes and quality of life through customized treatments. For positive tumor cells and moderately or severely abnormal cells, significant differences in genes arise as the disease occurs and progresses. Sequencing of DNA derived from urine had 82.2% sensitivity and 100% specificity, which may have great potential for the diagnosis of UUT-UC (73). In addition, in pathological cancer states, abnormal activation of export machinery, such as exosomes (products of vesicular transport), results in excessive discharge of many vital proteins and misexpression of miRNAs or mRNA, which are molecules with great differences in size (74, 75). The analysis of DNA mutations, including MDM2, TP53, RAS, and FGFR3, is an effective tumor phenotyping tool in defining the molecular subtypes with discrete profiles of gene expression, histology of UUT-UC, and prognostic outcome of patients (73, 76). It is worth focusing on the key molecular processes and analyzing gene and protein expression systematically. We believe that successful exploration of cancer therapeutic targets requires an interdisciplinary understanding of the processes underlying these cellular activating or inhibiting mechanisms and how secreted biomolecules participate in cell–cell interactions in the tumor microenvironment.

## Data Availability Statement

The datasets generated for our case-control study are available on request to the corresponding authors. Additionally, publicly available datasets for meta-analysis in our study could be searched in the PubMed, Medline, Embase, Cochrane Library and Scopus databases.

## Ethics Statement

The studies involving human participants were reviewed and approved by the Ethical Review Board of the Second Affiliated Hospital of Dalian Medical University, the Ethical Review Board of the Affiliated Dalian Friendship Hospital of Dalian Medical University, and the Ethical Review Board of the Cancer Hospital of China Medical University. Written informed consent was obtained from the individual(s) for the publication of any potentially identifiable images or data included in this article.

## Author Contributions

The author contributions were evaluated by four criteria that were formulated by the guidelines of the International Committee of Medical Journal Editors. BF, YaH, and YW designed the research. BF, YuH, SW, QT, XY, MS, TC, YaH, YW, and ZL acquired the data. BF, SW, and QT analyzed and interpreted the clinical data of the patients. SW and YW performed and interpreted the pathology staining. BF, YuH, and YW reviewed the literature and drafted the manuscript. BF, YuH, QL, YaH, and ZL assisted with image interpretation and formatting and critically revised the manuscript. All authors contributed to the article and approved the submitted version.

## Funding

BF was supported by the National Natural Science Foundation of China (No. 31800787); the Medical Scientific Research Project of Dalian City (No. 1812038); the United Fund of the Second Hospital of Dalian Medical University and Dalian Institute of Chemical Physics, Chinese Academy of Sciences (No. UF-QN-202004); and the Dalian High-Level Talents Innovation Support Program (No. 2019RQ014). ZL was supported by the National Natural Science Foundation of China (No. 81772739). The sponsors had no role in the analysis and interpretation of the data or the manuscript preparation, review, or approval.

## Conflict of Interest

The authors declare that the research was conducted in the absence of any commercial or financial relationships that could be construed as a potential conflict of interest.

## Publisher’s Note

All claims expressed in this article are solely those of the authors and do not necessarily represent those of their affiliated organizations, or those of the publisher, the editors and the reviewers. Any product that may be evaluated in this article, or claim that may be made by its manufacturer, is not guaranteed or endorsed by the publisher.

## Abbreviations

IVR, intravesical recurrence; UUT-UC, upper urinary tract urothelial carcinoma; RNU, radical nephroureterectomy; IV-RFS, intravesical recurrence-free survival; ROC curve, receiver operating characteristic curve; C-index, concordance index; AJCC, American Joint Commission on Cancer; HR, hazard ratio; RR, risk ratio; CI, confidence interval.
